# Effects of Ceftriaxone on Glial Glutamate Transporters in Wistar Rats Administered Sequential Ethanol and Methamphetamine

**DOI:** 10.3389/fnins.2016.00427

**Published:** 2016-09-22

**Authors:** Yusuf S. Althobaiti, Fahad S. Alshehri, Atiah H. Almalki, Youssef Sari

**Affiliations:** ^1^Department of Pharmacology and Experimental Therapeutics, College of Pharmacy and Pharmaceutical Sciences, University of ToledoToledo, OH, USA; ^2^Department of Medicinal and Biological Chemistry, College of Pharmacy and Pharmaceutical Sciences, University of ToledoToledo, OH, USA

**Keywords:** methamphetamine, GLT-1, hyperthermia, ethanol gavage, xCT, GLAST

## Abstract

Methamphetamine (METH) is one of the psychostimulants that is co-abused with ethanol. Repeated exposure to high dose of METH has been shown to cause increases in extracellular glutamate concentration. We have recently reported that ethanol exposure can also increase the extracellular glutamate concentration and downregulate the expression of glutamate transporter subtype 1 (GLT-1). GLT-1 is a glial transporter that regulates the majority of extracellular glutamate. A Wistar rat model of METH and ethanol co-abuse was used to examine the expression of GLT-1 as well as other glutamate transporters such as cystine/glutamate exchanger (xCT) and glutamate aspartate transporter (GLAST). We also examined the body temperature in rats administered METH, ethanol or both drugs. We further investigated the effects of ceftriaxone (CEF), a β-lactam antibiotic known to upregulate GLT-1, in this METH/ethanol co-abuse rat model. After 7 days of either ethanol (6 g/kg) or water oral gavage, Wistar rats received either saline or METH (10 mg/kg i.p. every 2 h × 4), followed by either saline or CEF (200 mg/kg) posttreatment. METH administered alone decreased GLT-1 expression in the nucleus accumbens (NAc) and prefrontal cortex (PFC) and increased body temperature, but did not reduce either xCT or GLAST expression in ethanol and water-pretreated rats. Interestingly, ethanol and METH were found to have an additive effect on the downregulation of GLT-1 expression in the NAc but not in the PFC. Moreover, ethanol alone caused GLT-1 downregulation in the NAc and elevated body temperature compared to control. Finally, CEF posttreatment significantly reversed METH-induced hyperthermia, restored GLT-1 expression, and increased xCT expression. These findings suggest the potential therapeutic role of CEF against METH- or ethanol/METH-induced hyperglutamatergic state and hyperthermia.

## Introduction

Methamphetamine (METH) abusers frequently use alcohol with a higher risk of reaching alcohol intoxication (Furr et al., 2000). The prevalence of alcohol use disorder was found to be more than 75% among amphetamine-dependent subjects (Stinson et al., 2005). Exposure to a high dose of METH induces depletion of dopamine and serotonin at the nerve terminals (Ricaurte et al., 1980, 1982; Seiden et al., 1988; Hirata et al., 1995; Cass et al., 2006) and increases extracellular glutamate concentration in rat striatum (Nash and Yamamoto, 1992; Stephans and Yamamoto, 1994). Repeated exposure to higher dose of amphetamine has also been shown to increase extracellular glutamate concentration in the nucleus accumbens (NAc) and the ventral tegmental area (VTA) in rats (Xue et al., 1996). Although, it is known that repeated METH exposure can increase extracellular glutamate concentration, there is less known about its effect on glutamate transporters. In general, these transporters are responsible for clearing extracellular glutamate concentration to maintain glutamate homeostasis. Among these transporters, glutamate transporter 1 (GLT-1; human homolog is excitatory amino acid transporter 2, EAAT2) plays a major role in clearing the majority of the extracellular glutamate concentration (Ginsberg et al., 1995; Rothstein et al., 1995; Danbolt, 2001; Mitani and Tanaka, 2003). Importantly, chronic ethanol exposure was found to reduce GLT-1 expression (Alhaddad et al., 2014b; Aal-Aaboda et al., 2015; Goodwani et al., 2015) and increase extracellular glutamate concentration in the NAc (Ding et al., 2013; Das et al., 2015; Pati et al., 2016). Since repeated exposure to high dose of METH can increase extracellular glutamate concentration (Halpin et al., 2014), we investigated in this study for any potential additive effect of ethanol and METH exposure on GLT-1 expression as well as other glial glutamate transporters such as cystine/glutamate transporter (xCT) and glutamate aspartate transporter (GLAST) in the NAc and PFC. The NAc is a brain region that is involved in the rewarding and reinforcing effects of drugs of abuse (Koob and Bloom, 1988; Wise and Rompré, 1989; Bardo, 1998; Koob et al., 1998). The NAc receives glutamatergic inputs from the PFC as well as other brain regions (Kelley et al., 1982; Phillipson and Griffiths, 1985). In this study, we examined the effect of ceftriaxone (CEF) posttreatment on GLT-1, xCT, and GLAST expression in the NAc and PFC in rats that were exposed to repeated high-dose METH. CEF is known to increase GLT-1 expression in several brain regions (Miller et al., 2008; Sari et al., 2009, 2013) and can normalize extracellular glutamate concentration in the NAc in cocaine and ethanol-seeking rat models (Trantham-Davidson et al., 2012; Das et al., 2015). CEF was also shown to reduce ethanol intake and cocaine seeking, in part, through upregulation of GLT-1 and xCT expression in the NAc and PFC (Sari et al., 2009, 2011; Knackstedt et al., 2010; Fischer et al., 2013; Alhaddad et al., 2014a; Rao and Sari, 2014). It is noteworthy that repeated exposure to high dose of METH was found to cause hyperthermia (Chan et al., 1994; Lan et al., 1998; Ishigami et al., 2003). Importantly, CEF was also revealed to reduce morphine-induced hyperthermia (Rawls et al., 2007). Thus, we have investigated the effects of CEF on METH-induced hyperthermia. We administered CEF after ethanol and METH exposure for clinical relevance.

## Materials and methods

### Subjects

Male Wistar rats, weighing 200–300 g at the beginning of the study, were obtained from Harlan, Inc. (Indianapolis, IN). Rats were single-housed in standard plastic cages with controlled temperature (21°C) and humidity (30%) on 12:12 light-dark cycle and were allowed to habituate to these conditions prior to the experiments. Rats had *ad libitum* food and water throughout the experimental procedure, except 2 h fasting prior to each oral gavage administration. Animal experimental procedures were approved by the Institutional Animal Care and Use Committee of The University of Toledo in accordance with the guidelines of the Institutional Animal Care and Use Committee of the National Institutes of Health and the Guide for the Care and Use of Laboratory Animals (Institute of Laboratory Animal Resources, Commission on Life Sciences, 1996).

### Drugs

(+) METH hydrochloride was purchased from Sigma-Aldrich (St. Louis, MO). CEF (Sandoz Inc., Princeton, NJ) was purchased from The University of Toledo's pharmacy. Saline solution (0.9% NaCl) was used to dissolve either (+) METH or CEF. Ethanol (95%; Decon Labs, Inc.) was diluted in water.

### Experimental design

**Figure 1:**
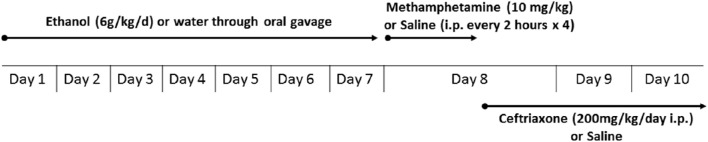
**Experimental schedule for METH and ethanol administration**. Rats were administered oral gavage of either water or ethanol (6 g/kg) for 7 days followed by either METH (10 mg/kg i.p., every 2 h for 4 times) or saline vehicle (i.p.). After completion of the four METH injections, rats were randomly assigned to receive either CEF (200 mg/kg i.p.) or saline vehicle (i.p.) for 2 days. Control and treated rats were then quickly euthanized (72 and 48 h following last water/ethanol and saline/METH administration, respectively) by CO2 inhalation and rapidly decapitated.

An experimental schedule is illustrated in Figure 1. Rats were administered oral gavage of either water or ethanol (6 g/kg) for 7 days, followed by either METH (10 mg/kg, i.p.) or saline vehicle (i.p.). We first orally gavaged the rats with ethanol in order to initially induce a reduction in GLT-1 expression and glutamate uptake, as it was performed in recent study from our laboratory (Das et al., 2015); we then followed with METH i.p. injections to further reduce glutamate uptake. After completion of the four METH i.p. injections, rats were randomly assigned to receive either CEF (200 mg/kg i.p.) or saline vehicle (i.p.) for 2 days; control and experimental groups have been summarized in Table 1. The rationale for testing repeated high dose of METH (10 mg/kg i.p. every 2 h × 4) exposure was chosen based on previous studies that showed neurotoxicity and elevation of extracellular glutamate concentration in rat brains (Bowyer et al., 1994; Hirata et al., 1995; Yamamoto and Zhu, 1998; Mark et al., 2004, 2007). The rationale for testing the ethanol binge gavage paradigm was based on recent studies from our laboratory and others (Faingold, 2008; Abulseoud et al., 2014; Das et al., 2016). Control and treated rats were then quickly euthanized by CO_2_ inhalation and rapidly decapitated. Brains were then extracted and immediately frozen in dry ice and stored at −80°C. The PFC and NAc were micropunched using a cryostat apparatus as described in a previous study from our laboratory (Sari and Sreemantula, 2012). Rat Brain Stereotaxic Atlas was used to identify the selected structures (PFC and NAc) (Paxinos and Watson, 2007).

**Table 1:** **Experimental groups according to the administration of water or ethanol oral gavage, METH or saline, as well as CEF or saline**.

**Group**	**Day 1–7**	**Day 8**		**Day 8–10**	
		**Drug**	**Dose**	**Drug**	**Dose**
1- Water-Saline-Saline	Water	Saline	(1 ml/kg, i.p. every 2 h × 4)	Saline	(1 ml/kg, i.p. every day × 3)
2- Water-METH-Saline	Water	METH	(10 mg/kg, i.p. every 2 h × 4)	Saline	(1 ml/kg, i.p. every day × 3)
3- Water-METH-CEF	Water	METH	(10 mg/kg, i.p. every 2 h × 4)	CEF	(200 mg/kg, i.p. every day × 3)
4- Ethanol-Saline-Saline	Ethanol	Saline	(1 ml/kg, i.p. every 2 h × 4)	Saline	(1 ml/kg, i.p. every day × 3)
5- Ethanol-METH-Saline	Ethanol	METH	(10 mg/kg, i.p. every 2 h × 4)	Saline	(1 ml/kg, i.p. every day × 3)
6- Ethanol-METH-CEF	Ethanol	METH	(10 mg/kg, i.p. every 2 h × 4)	CEF	(200 mg/kg, i.p. every day × 3)

### Western blot

The Western blot procedure was performed as previously described (Sari et al., 2009). Briefly, brain tissue was lysed in lysis buffer (50 mM Tris–HCl, 150 mM NaCl, 1 mM EDTA, 0.5% NP-40, 1% Triton, 0.1% SDS) containing a protease inhibitor cocktail. A Bio-Rad protein assay method was used to determine total protein content in the tissue extracts (Bio-Rad, Hercules, CA, USA). The extracted proteins were loaded onto 10–20% tris-glycine gel. After separation, proteins were transferred electrophoretically from the gel onto the PVDF membranes. The membranes were then blocked using 3% milk in Tris-buffered saline Tween 20 for 30 min. Guinea pig anti-GLT-1 (1:5000 dilution; Millipore Bioscience Research Reagents), rabbit anti-xCT antibody (1:1000 dilution: Novus), rabbit anti-GLAST (1:5000 dilution; Abcam), or mouse anti β-tubulin antibody (1:5000 dilution; Covance) was then added to the blocking buffer, and the membrane was incubated overnight at 4°C. The membrane was then washed and incubated with horseradish peroxidase-labeled (HRP) anti-Guinea pig, anti-rabbit, or anti-mouse secondary antibody (1:5000). A chemiluminescent kit (SuperSignal West Pico) was used to incubate the membrane for protein detection. Subsequently, the membrane was exposed to Kodak BioMax MR films (Thermo Fisher Scientific). The films were then developed using an SRX-101A machine by Konica Minolta Medical & Graphic, Inc. The blots for each protein were digitized, and densitometric analysis was obtained using an MCID software (Imaging Research, Inc.). Data were calculated as ratios of GLT-1/β-tubulin, xCT/β-tubulin, and GLAST/β-tubulin. The control group (Water-Saline-Saline) was included with the drug treatment groups each time the 10-well gel was run. The control group was set arbitrary as 100% and the changes in protein expression of the remaining five groups were obtained relative to the control group in that particular gel. The expression of proteins was consistent between control and drug treatment groups (six groups) in each 10-well gel. This calculation method has been used in several studies from ours and others (Li et al., 2003; Raval et al., 2003; Miller et al., 2008; Zhang and Tan, 2011; Simões et al., 2012; Devoto et al., 2013; Goodwani et al., 2015; Hakami et al., 2016).

### Body temperature measurement

The body temperature was measured rectally using digital thermometer (Thermalert TH-5, Physitemp, NJ, USA) at three time points to minimize handling following METH exposure: at baseline, after the last METH injection (Time 0) when the rats were randomly assigned to receive either saline or CEF, and finally 12 h after last METH injection.

### Statistical analysis

Two-way ANOVA (Pretreatment × Posttreatment) was used to analyze immunoblot data. Newman-Keuls multiple comparisons test was used when significant interaction or significant main effect was revealed using GraphPad Prism. Mixed-model factorial ANOVA [Time × Pretreatment × Posttreatment, with repeated measures on the time factor (Baseline, 0, 12 h), with Pretreatment and Posttreatment as the between-subjects factor] was used to analyze body temperature data using SPSS software. All statistical tests were based on *p* < 0.05 level of significance.

## Results

### Effects of METH administered alone or with ethanol as well as effects of CEF posttreatment on GLT-1 expression in the NAc and PFC

**Figure 2:**
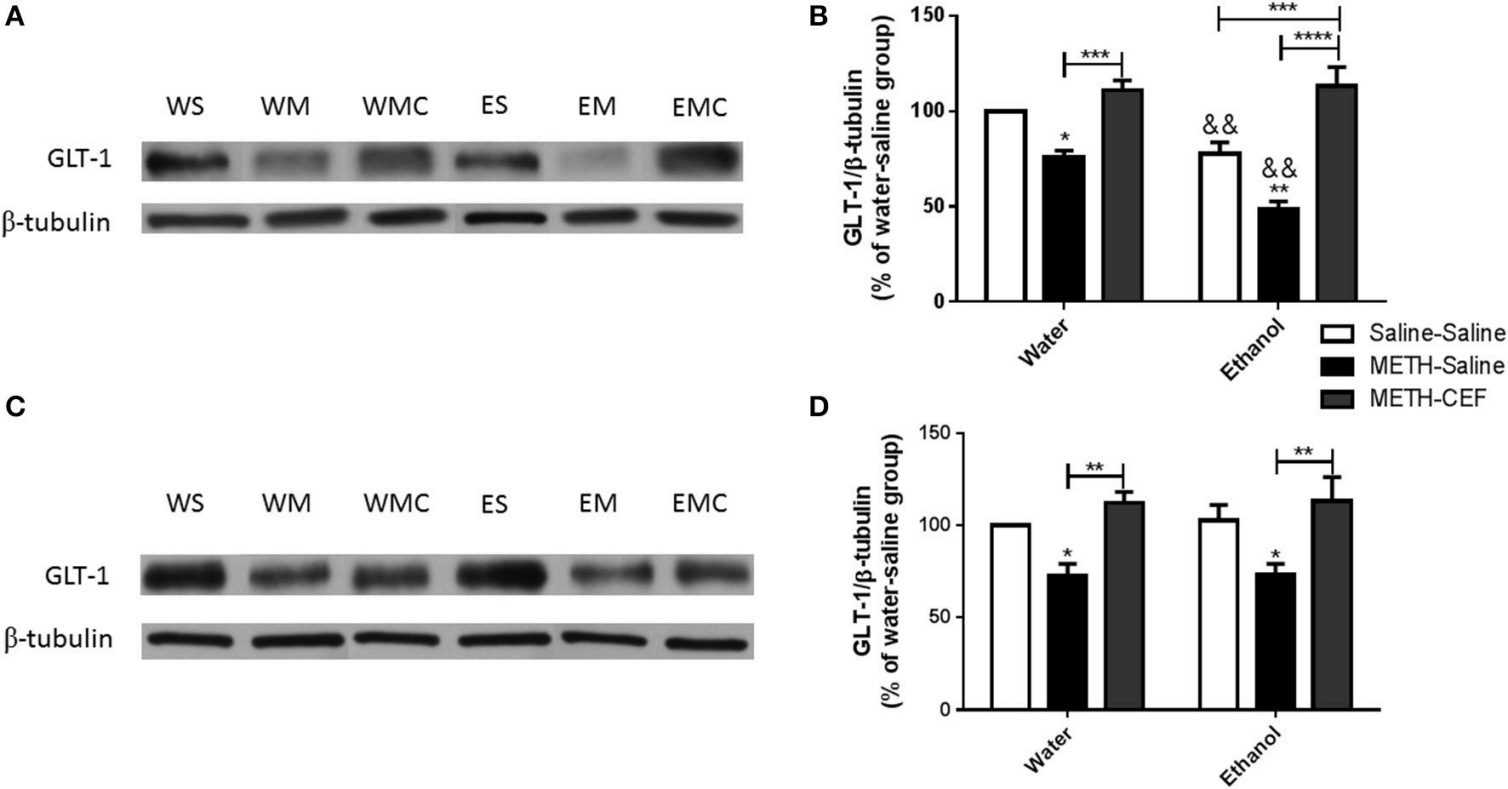
**Effects of METH (10 mg/kg i.p. every 2 h × 4), ethanol and CEF (200 mg/kg) on GLT-1 expression in the NAc and PFC. (A,C)** Immunoblots for GLT-1 as well as β-tubulin, which was used as a control loading protein, in the NAc and PFC, respectively, as compared to water-pretreated groups and ethanol-pretreated groups. **(B,D)** Quantitative analysis revealed a significant increase in the ratio of GLT-1/β-tubulin in METH-CEF-treated (WMC or EMC) rats compared to the METH-Saline-treated rats in the water (WM) and the ethanol (EM) groups, in the NAc and PFC, respectively. Significant downregulation of GLT-1 expression was revealed in the METH-Saline-treated groups compared to control in water- and ethanol-treated groups in the NAc and PFC. Significant downregulation of GLT-1 expression was revealed in ethanol-Saline-Saline (ES) and ethanol-METH-Saline (EM) groups compared to its corresponding water control groups in the NAc, but not in the PFC. No significant difference in GLT-1 expression was revealed in water-METH-CEF-treated (WMC) rats compared to water control groups. However, a significant increase in GLT-1 expression was found in the Ethanol-METH-CEF (EMC) group compared to ethanol control (ES) group in the NAc, but not in the PFC. ^*^p < 0.05, ^**^p < 0.01 (or &&, for comparison between ethanol and its corresponding water control groups), ^***^p < 0.001, and ^****^p < 0.0001. Values shown as means ± S.E.M. *n* = 6 for each group.

This study investigated the effect of METH on GLT-1 expression in the NAc and PFC 48 h following the last METH i.p. injection in Wistar rats. Two-way ANOVA revealed a significant effect of posttreatment in the NAc [*F*_(2, 30)_ = 39.09, *p* < 0.0001] and PFC [*F*_(2, 30)_ = 14.10, *p* < 0.0001], significant effect of oral gavage pretreatment in the NAc [*F*_(1, 30)_ = 11.69, *p* < 0.0018] but not PFC [*F*_(1, 30)_ = 0.05634, *p* = 0.8140], and significant interaction between posttreatment and oral gavage pretreatment in the NAc [*F*_(2, 30)_ = 3.949, *p* = 0.0300] but not in the PFC [*F*_(2, 30)_ = 0.009251, *p* = 0.9908]. Newman-Keuls multiple comparisons test showed a significant increase in GLT-1 expression in METH-CEF-treated rats compared to METH-Saline-treated rats in the NAc [water group (*p* < 0.001) and ethanol group (*p* < 0.0001; Figures 2A,B)] and PFC [water group (*p* < 0.01) and ethanol group (*p* < 0.01; Figures 2C,D)]. Moreover, statistical analyses showed a significant downregulation of GLT-1 expression in the NAc [water group (*p* < 0.05) and ethanol group (*p* < 0.01; Figure 2B)] and in the PFC [water group (*p* < 0.05) and ethanol group (*p* < 0.05; Figure 2D)] of the METH-Saline group compared to the corresponding saline control group. Alternatively, *post-hoc* analyses showed a significant decrease in GLT-1 expression in Ethanol-Saline-Saline compared to Water-Saline-Saline in the NAc (*p* < 0.01; Figure 2B). Interestingly, GLT-1 expression was significantly decreased in Ethanol-METH-Saline-treated rats compared to Water-METH-Saline-treated rats in the NAc (*p* < 0.01; Figure 2B).

### Effects of CEF treatment on xCT expression in the NAc and PFC of groups administered METH alone or METH and ethanol

**Figure 3:**
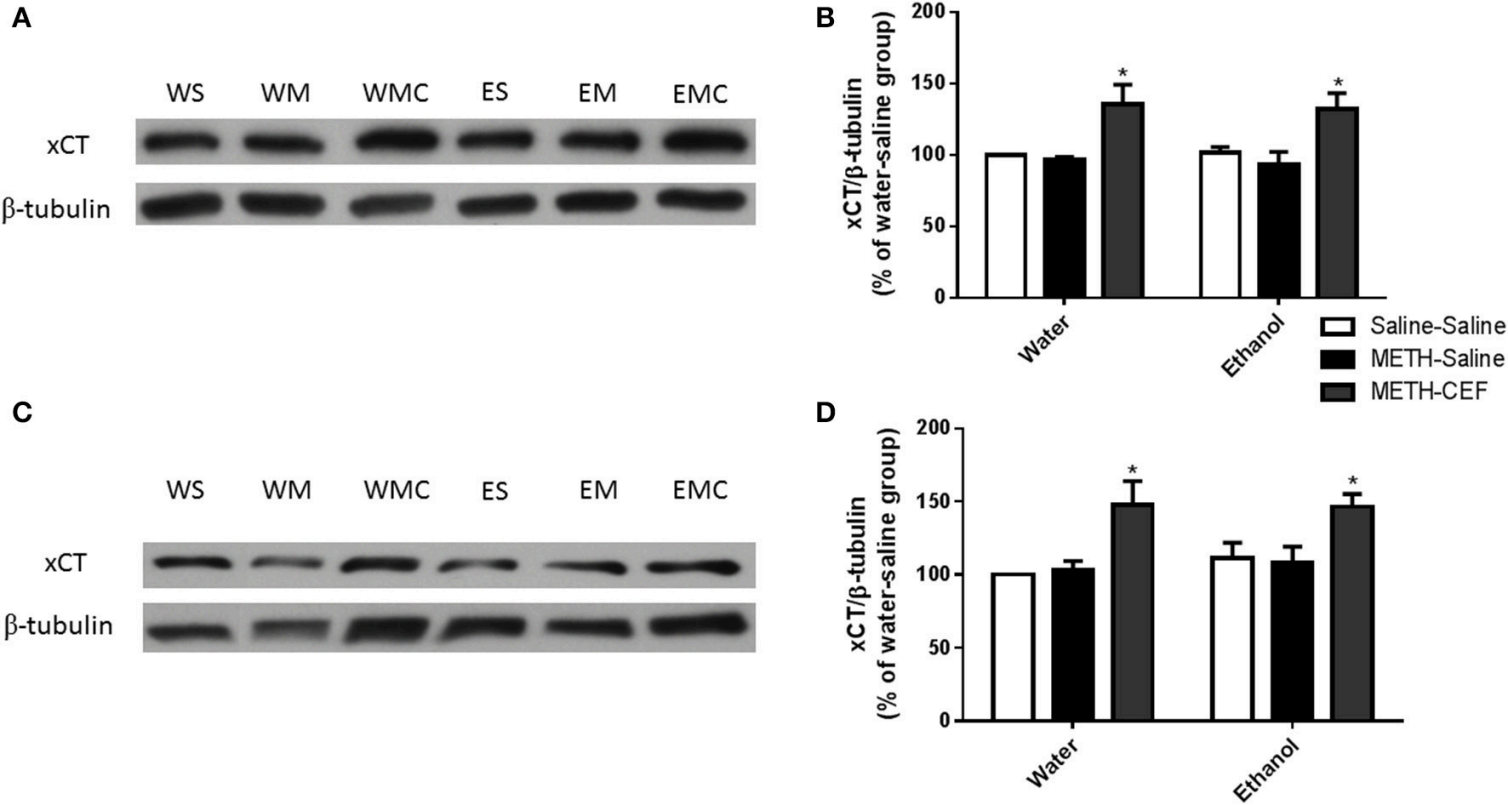
**Effects of METH (10 mg/kg i.p. every 2 h × 4), ethanol and CEF (200 mg/kg) on xCT expression in the NAc and PFC. (A,C)** Immunoblots for xCT as well as β-tubulin, which was used as a control loading protein, in the NAc and PFC, respectively, as compared to water-pretreated groups and ethanol-pretreated groups. **(B,D)** Quantitative analysis revealed a significant increase in the ratio of xCT/β-tubulin in METH-CEF-treated rats compared to the METH-Saline and Saline-Saline treated rats in the water and ethanol groups in the NAc and PFC, respectively. No significant change in xCT expression was revealed in the METH-Saline-treated groups compared to control in water- and ethanol-treated groups in either the NAc or PFC. ^*^p < 0.05. Values shown as means ± S.E.M. *n* = 6 for each group.

We further investigated the effect of METH on xCT expression in the NAc and PFC 48 h following the last METH i.p. injection in Wistar rats. Two-way ANOVA revealed a significant effect of posttreatment in the NAc [*F*_(2, 30)_ = 12.92, *p* < 0.0001] and PFC [*F*_(2, 30)_ = 11.01, *p* < 0.001], no significant effect of oral gavage pretreatment in the NAc [*F*_(1, 30)_ = 0.04864, *p* = 0.8269] or PFC [*F*_(1, 30)_ = 0.3730, *p* = 0.5460], and no significant interaction between posttreatment and oral gavage pretreatment in the NAc [*F*_(2, 30)_ = 0.05490, *p* = 0.9467] or PFC [*F*_(2, 30)_ = 0.1945, *p* = 0.8243]. Newman-Keuls multiple comparisons test showed a significant increase in xCT expression in METH-CEF-treated rats compared to METH-Saline and Saline-Saline treated rats in the NAc [water group (*p* < 0.05) and ethanol group (*p* < 0.05; Figures 3A,B)] and PFC [water group (*p* < 0.05) and ethanol group (*p* < 0.05; Figures 3C,D)]. However, statistical analyses did not show any significant change in xCT expression in the NAc [in water group (*p* > 0.05) or ethanol group (*p* > 0.05; Figure 3B)] and PFC [water group (*p* > 0.05) or ethanol group (*p* > 0.05; Figure 3D)] of the METH-Saline group compared to the corresponding saline control group.

### Effects of CEF treatment in GLAST expression in the NAc and PFC in groups administered METH alone or with ethanol

**Figure 4:**
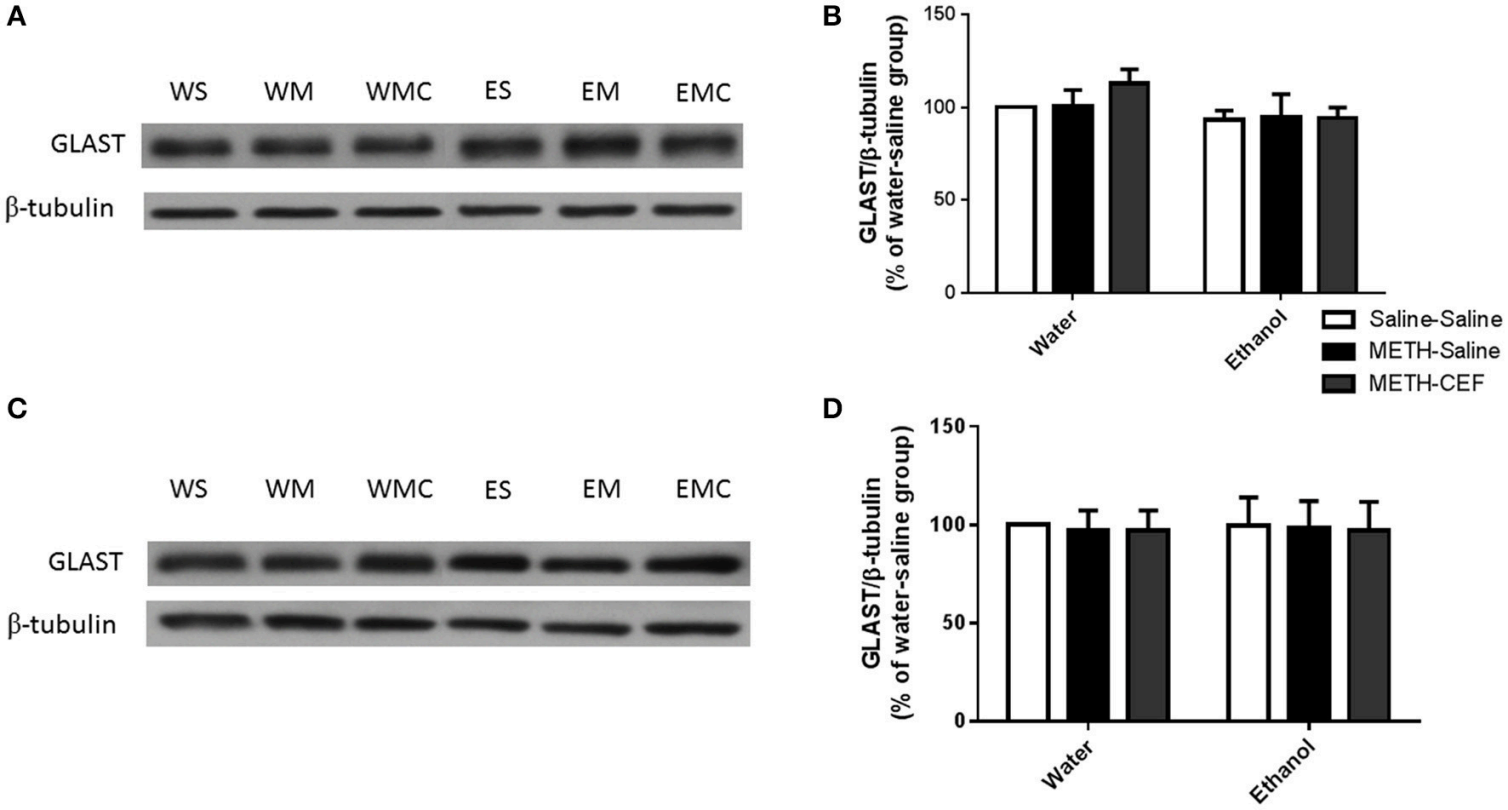
**Effects of METH (10 mg/kg i.p. every 2 h × 4), ethanol and CEF (200 mg/kg) on GLAST expression in the NAc and PFC. (A,C)** Immunoblots for GLAST as well as β-tubulin, which was used as a control loading protein, in the NAc and PFC, respectively, as compared to water-pretreated groups and ethanol-pretreated groups. **(B,D)** Quantitative analysis did not reveal any significant differences in the ratio of GLAST/β-tubulin among all groups in the NAc and PFC, respectively. Values shown as means ± S.E.M. *n* = 6 for each group.

We further investigated the effect of METH on GLAST expression in the NAc and PFC. Two-way ANOVA did not reveal any significant effect of posttreatment in the NAc [*F*_(2, 30)_ = 0.4872, *p* = 0.6191] or PFC [*F*_(2, 30)_ = 0.02371, *p* = 0.9766], no significant effect of oral gavage pretreatment in the NAc [*F*_(1, 30)_ = 2.810, *p* = 0.1041] and PFC [*F*_(1, 30)_ = 0.0008578, *p* = 0.9768], and no significant interaction between posttreatment and oral gavage pretreatment in the NAc [*F*_(2, 30)_ = 0.4643, *p* = 0.6330] (Figures 4A,B) and PFC [*F*_(2, 30)_ = 0.003179, *p* = 0.9968] (Figures 4C,D).

### Effect of CEF on METH-induced hyperthermia

**Figure 5:**
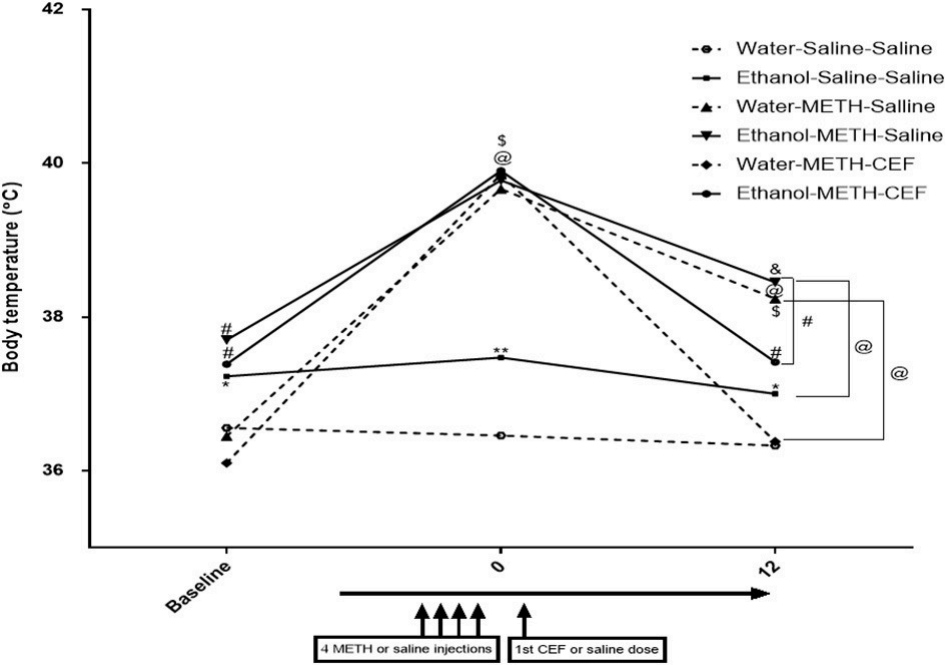
**Effects of METH (10 mg/kg i.p. every 2 h × 4), ethanol, and CEF posttreatment (200 mg/kg, i.p.) on body temperature compared to Water-Saline-Saline control group**. Ethanol pretreatment significantly elevated body temperature at baseline compared to water pretreatment. METH significantly elevated body temperature in all groups after the last METH injection (time 0) compared to Water-Saline-Saline group. CEF posttreatment restored body temperature compared to saline posttreatment in water and ethanol-METH treated rats. ^*^p < 0.05, ^**^p < 0.01, ^#^*p* < 0.001, and ^@^*p* < 0.0001 (^&^*p* < 0.01, ^$^*p* < 0.001 compared to baseline point) (mixed-model repeated measure factorial ANOVA). Values are represented as mean ± SEM (Error bars were deleted for clarity). *n* = 7–9 for each group.

A mixed-model factorial ANOVA conducted on body temperature revealed a significant effect of time [*F*_(2, 39)_ = 240.305, *p* < 0.0001], a significant interaction between time and pretreatment [*F*_(2, 39)_ = 7.848, *p* = 0.001], a significant interaction between time and posttreatment [*F*_(4, 80)_ = 33.22, *p* < 0.0001], and a significant interaction between time, pretreatment and posttreatment [*F*_(4, 80)_ = 4.335, *p* = 0.003]. Contrast analyses revealed that ethanol pretreatment significantly elevated body temperature compared to water control at baseline (*p* < 0.05) (Figure 5). Similarly, contrast analyses showed that following the last dose of METH (at time 0), METH significantly elevated body temperature compared to saline in water and ethanol pretreated groups as well as in comparison to the baseline point (*p* < 0.0001). In addition, body temperature was significantly higher in the ethanol-Saline-Saline group as compared to the Water-Saline-Saline group at this time point (*p* < 0.001). CEF posttreatment (at time 12 h) restored body temperature compared to saline in the water (*p* < 0.0001) and ethanol (*p* < 0.001) pretreated groups. Similarly, body temperature was significantly higher in the water-METH-Saline (*p* < 0.0001), ethanol-Saline-Saline (*p* < 0.05), Ethanol-METH-Saline (*p* < 0.0001), and Ethanol-METH-CEF groups (*p* < 0.01) as compared to the Water-Saline-Saline (Figure 5) group. Significant increase in body temperature was revealed in METH-Saline treated groups in water and ethanol pretreatment groups as compared to its baseline point. No significant difference was found between Water-METH-CEF and Water-Saline-Saline groups or between Ethanol-METH-CEF and Ethanol-Saline-Saline groups (*p* > 0.05) (Figure 5).

## Discussion

The present study revealed for the first time that repeated high dose of METH significantly decreased GLT-1 expression in the NAc and PFC in the co-abuse METH and ethanol group as well as in the METH alone group. Our findings contradict a previous report in which METH induced an upregulation of GLT-1 in the PFC (Qi et al., 2012). However, this contradictory result could be due to different experimental designs and dosing regimens. A single low dose of METH (2 mg/kg), as compared to 10 mg/kg every 2 h for 4 times in this study, was used in the previous report by Qi et al. (2012). Mice were then euthanized at different time points following a single METH injection (0.5, 1, 2, and 4 h) compared to rats that were euthanized 48 h after the last METH injection in our report. The GLT-1 expression was not changed in the PFC at the first two time points tested. However, GLT-1 expression was then increased after 2 h of METH injection (~250% of control) and then decreased dramatically to reach 140% of control after 4 h of METH injection. This previous report did not further investigate GLT-1 expression beyond 4 h of METH administration. The pattern of changes in GLT-1 expression presented in this previous report suggests a transient increase of GLT-1 that might be followed by a reduction in GLT-1 expression. By contrast, in our study, we have used repeated high dose METH, which is well known to produce neurotoxicity and hyperthermia (Sonsalla et al., 1989; Bowyer et al., 1994; Halpin and Yamamoto, 2012). This neurotoxic dosing paradigm of METH produced a comparable neurotoxicity to other studies that have used a very high dose of METH 50 mg/kg 2–3 times per day for 4 days (Bittner et al., 1981; Ricaurte et al., 1982). However, previous study has investigated a single dose of METH in order to produce neurotoxicity and hyperthermia comparable to the dosing paradigm used in this study (Fukumura et al., 1998). The least effective dose that produced neurotoxicity and hyperthermia was 10 times higher than the dose used by Qi et al. (2012) (i.e., 20 mg/kg), while doses that produced a comparable neurotoxicity and hyperthermia are 30 and 40 mg/kg (Fukumura et al., 1998; Qi et al., 2012).

The GLT-1 is a glial glutamate transporter that plays a critical role in clearing the majority of extracellular glutamate to maintain glutamate homeostasis (Ginsberg et al., 1995; Rothstein et al., 1995, 1996; Danbolt, 2001; Mitani and Tanaka, 2003). The METH-induced downregulation of GLT-1 expression could be due to the fact that binge METH exposure induced hepatotoxicity in rats, with subsequent elevation in plasma and brain ammonia (Halpin and Yamamoto, 2012). Chronic ammonia exposure for at least 48 h was shown to decrease glutamate uptake in cultured astrocytes due to a possible decrease in the expression of glutamate transporters (Bender and Norenberg, 1996). It has also been shown that ammonia is responsible for GLT-1 downregulation in brains of a rat model with acute liver failure (Knecht et al., 1997; Chan and Butterworth, 1999). Moreover, rats treated with ammonium acetate develop a significant downregulation in GLT-1 expression compared to control rats (Norenberg et al., 1997). Therefore, downregulation of GLT-1 expression found in this study could be due to elevation in plasma and brain ammonia caused by METH exposure. Further studies are warranted to determine the concentration of ammonia in the plasma and brain of rats exposed to repeated doses of METH.

A METH-induced decrease in GLT-1 expression in the NAc and PFC was restored by CEF posttreatment in both ethanol and water-pretreated rats. CEF is known to upregulate GLT-1 expression in disease and naïve animal models (Rothstein et al., 2005; Miller et al., 2008; Ramos et al., 2010). Since METH caused GLT-1 downregulation, we further investigated GLT-1 expression in ethanol and water pretreated rats to explore whether there is any additive effect of ethanol and METH in this protein. The present data revealed that METH exacerbates the reduction in GLT-1 expression in ethanol-pretreated rats compared to water-pretreated rats in the NAc, but not in the PFC. This indicates that there is no additive effect of ethanol on GLT-1 expression in the PFC, which is consistent with our recent findings demonstrated that free choice exposure to ethanol does not reduce GLT-1 expression in the PFC (Sari et al., 2013; Alhaddad et al., 2014b). Moreover, GLT-1 expression was downregulated in the NAc following saline treatment in the ethanol group compared to the water group. This is in accordance with recent findings demonstrated that chronic ethanol exposure decreases GLT-1 expression and increases extracellular glutamate concentration in the NAc (Das et al., 2015).

Although the mechanism of ethanol-induced downregulation of GLT-1 is not known, studies from our laboratory showed that ethanol decreases phosphorylation of Akt (Alhaddad et al., 2014b; Goodwani et al., 2015). Certain studies, however, have reported contradicting findings regarding the effects of ethanol exposure on GLT-1 expression. For example, GLT-1 expression was not altered following intermittent ethanol exposure (Pati et al., 2016) or continuous ethanol exposure for 8 weeks in female P rats (Ding et al., 2013). These contradictory results could be due to the differences in ethanol exposure paradigm and study design. The report by Pati et al. (2016) used intermittent ethanol exposure, while in our present study, we have used repeated daily ethanol exposure, which has been shown to increase extracellular glutamate concentration shortly after the last ethanol exposure in different brain regions such as the VTA, hippocampus, NAc, PFC, and striatum (Rossetti and Carboni, 1995; Dahchour and Witte, 1999; Dahchour and De Witte, 2000; Melendez et al., 2005; Kapasova and Szumlinski, 2008; Ding et al., 2012; Hermann et al., 2012) and decrease GLT-1 expression and/or glutamate clearance (Melendez et al., 2005; Ding et al., 2012; Aal-Aaboda et al., 2015; Das et al., 2015; Goodwani et al., 2015). Alternatively, the report by Ding et al. (2013) used free choice continuous ethanol exposure for 8 weeks (compared to oral gavage of ethanol for 7 days in this current study). Ding et al. noted a trend of decrease in GLT-1 expression that was suggested to be masked by high variations in samples. Moreover, this previous report used female rats, as opposed to male rats that showed a decrease in GLT-1 expression following ethanol exposure (Alhaddad et al., 2014b; Goodwani et al., 2015). Further studies are needed to investigate different gender responses to ethanol exposure and consequent changes in GLT-1 expression.

We also tested xCT, a glial protein that exchanges intracellular glutamate for extracellular cystine to maintain glutamate homeostasis (Bannai and Kitamura, 1980; Baker et al., 2002). However, we did not find any downregulation of xCT expression following METH treatment in either ethanol or water-pretreated rats. Importantly, CEF upregulated xCT expression in the NAc and PFC of ethanol and water pretreated rats, which is consistent with studies from our laboratory and others (Lewerenz et al., 2009; Knackstedt et al., 2010; Alhaddad et al., 2014a; Rao and Sari, 2014). CEF-induced upregulation of xCT expression might be another mechanism that modulates glutamate homeostasis to alleviate METH effects. In addition, xCT has been shown to facilitate cystine uptake with the subsequent synthesis of glutathione (Sato et al., 1999; Lewerenz et al., 2006). An *in vitro* study showed that CEF-induced upregulation of xCT expression was associated in part with increased glutathione concentration, which is independent of GLT-1 upregulation (Lewerenz et al., 2009). It is noteworthy that several studies indicated that METH can cause oxidative stress in different brain regions (Cubells et al., 1994; Açikgöz et al., 1998; Yamamoto and Zhu, 1998; Gluck et al., 2001; Ramirez et al., 2009). Furthermore, glutathione was found to be reduced in the striatum following repeated high doses of METH (Moszczynska et al., 1998). As a result, the CEF-induced increase in xCT expression may eventually improve glutathione synthesis.

Furthermore, we did not find any changes in GLAST expression in the NAc and PFC in either ethanol or water-pretreated rats. In accordance, ethanol exposure and/or CEF treatment did not significantly reduce GLAST expression (Alhaddad et al., 2014b; Hakami et al., 2016). Studies suggested that GLAST is highly expressed in the cerebellum and predominantly regulates glutamate uptake as compared to forebrain regions, including the PFC and NAc. However, GLT-1 is predominant in the forebrain. The differential predominance of GLAST vs. GLT-1 in the PFC and NAc might be a key factor involving the effects of ethanol and METH co-abuse in the expression of these transporters. Studies are warranted to investigate the differential effects of these glial transporters in an ethanol and METH co-abuse model.

METH significantly elevated body temperature in both ethanol and water-pretreated rats compared to saline, which is consistent with previous reports (Cass et al., 2006; Shioda et al., 2010; Halpin and Yamamoto, 2012). The present data showed that CEF posttreatment significantly reversed the increase in body temperature compared to saline when measured 12 h following the last METH dose. Although the rapid onset of action of CEF on body temperature is unclear, studies demonstrated that a single dose of CEF can increase the activity of GLT-1 and improve the survival of neurons (Thöne-Reineke et al., 2008). In fact, acute administration of CEF has many effects, including an anti-inflammatory response and analgesic action (Wei et al., 2012; Macaluso et al., 2013). Further studies are warranted to investigate the acute effects of CEF on the glutamatergic system and body temperature. The mechanism of action of CEF in reversing hyperthermia is unknown, but is most likely through its ability to upregulate GLT-1 and improve glutamate uptake. This is in line with a previous report by Rawls and colleagues in which CEF reversed morphine-induced hyperthermia (Rawls et al., 2007). This latter study demonstrates that CEF's inhibition of hyperthermia was prevented by administering glutamate uptake blocker (TBOA), which suggests that upregulation of GLT-1 expression may be critical in the attenuation of hyperthermia. It is unclear whether the normalizing effect of CEF on body temperature might be associated with upregulation of GLT-1 and reduced extracellular glutamate concentration in central reward brain regions such as the NAc and PFC. However, it is suggested that glutamate might be implicated in thermoregulation, since treatment with glutamate receptor antagonists attenuates the increase in body temperature in animal models (Madden and Morrison, 2003; Nakamura et al., 2004; Cao and Morrison, 2006; Nakamura and Morrison, 2008). Importantly, the NAc and PFC were found to be implicated in thermoregulation (Tseng et al., 1980; Hori et al., 1984; Shibata et al., 1988). Changes in body temperature and heat production were also found when functional ablation of PFC was applied (Shibata et al., 1981, 1985). In addition, a recent study has shown that microinjections of METH into the PFC evoked measures of non-shivering thermogenesis (Hassan et al., 2015). Further studies are warranted to explore any possible associative effects between thermoregulation and glutamate homeostasis in an ethanol and METH co-abuse animal model and to investigate the key brain regions involved in this mechanism.

In summary, our findings provide evidence of the important role of GLT-1 using high dose of METH, well known to cause a hyperglutamatergic state and hyperthermia. Importantly, we found for the first time additive effects of ethanol and METH on GLT-1 downregulation in the NAc as compared to drug administered alone. This study also showed for the first time that CEF, a β-lactam antibiotic, was effective in restoring GLT-1 expression and reversing hyperthermia in the ethanol and METH co-abuse rat model. These findings suggest that CEF might be used as a potential drug for treatment against METH- or ethanol/METH-induced downregulation of GLT-1 expression and hyperthermia.

## Author contributions

YA—Participated in study design and conceptualization, collected and analyzed data, helped with data interpretation, and drafted the manuscript. FA—Helped with data collection, analysis, and interpretation, and approved the final version of the manuscript. AA—Helped with data collection and approved the final version of the manuscript. YS—Conceptualized and designed the study, revised the manuscript for intellectual content, and approved the final version.

### Conflict of interest statement

The authors declare that the research was conducted in the absence of any commercial or financial relationships that could be construed as a potential conflict of interest.
